# The GenTree Dendroecological Collection, tree-ring and wood density data from seven tree species across Europe

**DOI:** 10.1038/s41597-019-0340-y

**Published:** 2020-01-02

**Authors:** Elisabet Martínez-Sancho, Lenka Slámová, Sandro Morganti, Claudio Grefen, Barbara Carvalho, Benjamin Dauphin, Christian Rellstab, Felix Gugerli, Lars Opgenoorth, Katrin Heer, Florian Knutzen, Georg von Arx, Fernando Valladares, Stephen Cavers, Bruno Fady, Ricardo Alía, Filippos Aravanopoulos, Camilla Avanzi, Francesca Bagnoli, Evangelos Barbas, Catherine Bastien, Raquel Benavides, Frédéric Bernier, Guillaume Bodineau, Cristina C. Bastias, Jean-Paul Charpentier, José M. Climent, Marianne Corréard, Florence Courdier, Darius Danusevicius, Anna-Maria Farsakoglou, José M. García del Barrio, Olivier Gilg, Santiago C. González-Martínez, Alan Gray, Christoph Hartleitner, Agathe Hurel, Arnaud Jouineau, Katri Kärkkäinen, Sonja T. Kujala, Mariaceleste Labriola, Martin Lascoux, Marlène Lefebvre, Vincent Lejeune, Mirko Liesebach, Ermioni Malliarou, Nicolas Mariotte, Silvia Matesanz, Tor Myking, Eduardo Notivol, Birte Pakull, Andrea Piotti, Mehdi Pringarbe, Tanja Pyhäjärvi, Annie Raffin, José A. Ramírez-Valiente, Kurt Ramskogler, Juan J. Robledo-Arnuncio, Outi Savolainen, Silvio Schueler, Vladimir Semerikov, Ilaria Spanu, Jean Thévenet, Mari Mette Tollefsrud, Norbert Turion, Dominique Veisse, Giovanni Giuseppe Vendramin, Marc Villar, Johan Westin, Patrick Fonti

**Affiliations:** 1 Swiss Federal Institute for Forest Snow and Landscape Research WSL, Zürcherstrasse 111, 8903 Birmensdorf, Switzerland; 2 Philipps-Universität Marburg, Department of Biology, Karl-von-Frisch-Strasse 8, 35043 Marburg, Germany; 3 Department of Biogeography and Global Change, National Museum of Natural Sciences MNCN-CSIC, Serrano 115 dpdo, 28006 Madrid, Spain; 4 Bavarian Office for Forest Seeding and Planting – ASP, Forstamtsplatz 1, 83317 Teisendorf, Germany; 5 Centre for Ecology and Hydrology NERC, EH26 0QB Edinburgh, United Kingdom; 6 Institut National de la Recherche Agronomique (INRA), Domaine Saint Paul, Site Agroparc, 84914 Avignon, France; 7 Instituto Nacional de Investigación y Tecnología Agraria y Alimentaria - Centro de Investigación Forestal (INIA-CIFOR), Ctra. de la Coruña km 7.5, 28040 Madrid, Spain; 8 Laboratory of Forest Genetics and Tree Breeding, School of Forestry and Natural Environment, Aristotle University of Thessaloniki, 54124 Thessaloniki, Greece; 9 Institute of Biosciences and BioResources, National Research Council (CNR), via Madonna del Piano 10, 50019 Sesto Fiorentino, Italy; 10 Institut National de la Recherche Agronomique (INRA), Avenue de la Pomme de pin 2163, 45075 Orléans, France; 11 Institut National de la Recherche Agronomique (INRA), route d’Arcachon 69, 33610 Cestas, France; 12 Vytautas Magnus University, Studentu Street 11, 53361 Akademija, Lithuania; 13 LIECO GmbH & Co KG, Forstgarten 1, 8775 Kalwang, Austria; 14 Natural Resources Institute Finland, University of Oulu, Paavo Havaksen tie 3, 90014 Oulu, Finland; 15 Department of Ecology & Genetics, EBC, Uppsala University, Norbyvägen 18D, 75236 Uppsala, Sweden; 16 Thünen Institute of Forest Genetics, Sieker Landstr. 2, 22927 Grosshansdorf, Germany; 17 Área de Biodiversidad y Conservación, Universidad Rey Juan Carlos, Calle Tulipán s/n, 28933 Móstoles, Spain; 18 Division of Forestry and Forest Resources, Norwegian Institute of Bioeconomy Research (NIBIO), P.O. Box 115, 1431 Ås, Norway; 19 Centro de Investigación y Tecnología Agroalimentaria de Aragón - Unidad de Recursos Forestales (CITA), Avda. Montañana 930, 50059 Zaragoza, Spain; 20 University of Oulu, Pentti Kaiteran katu 1, 90014 Oulu, Finland; 21 Austrian Research Centre for Forests (BFW), Seckendorff-Gudent-Weg 8, 1131 Wien, Austria; 22 Institute of Plant and Animal Ecology, Ural branch of RAS, 8 Marta St. 202, 620144 Ekaterinburg, Russia; 23 Skogforsk, Tomterna 1, 91821 Sävar, Sweden

**Keywords:** Forestry, Forest ecology

## Abstract

The dataset presented here was collected by the GenTree project (EU-Horizon 2020), which aims to improve the use of forest genetic resources across Europe by better understanding how trees adapt to their local environment. This dataset of individual tree-core characteristics including ring-width series and whole-core wood density was collected for seven ecologically and economically important European tree species: silver birch (*Betula pendula*), European beech (*Fagus sylvatica*), Norway spruce (*Picea abies*), European black poplar (*Populus nigra*), maritime pine (*Pinus pinaster*), Scots pine (*Pinus sylvestris*), and sessile oak (*Quercus petraea*). Tree-ring width measurements were obtained from 3600 trees in 142 populations and whole-core wood density was measured for 3098 trees in 125 populations. This dataset covers most of the geographical and climatic range occupied by the selected species. The potential use of it will be highly valuable for assessing ecological and evolutionary responses to environmental conditions as well as for model development and parameterization, to predict adaptability under climate change scenarios.

## Abstract (summary)

**Table:** 

Measurement(s)	growth ring • wood density
Technology Type(s)	measuring table • calculation
Factor Type(s)	tree species
Sample Characteristic - Organism	Betula pendula • Fagus sylvatica • Picea abies • Populus nigra • Pinus pinaster • Pinus sylvestris • Quercus petraea
Sample Characteristic - Location	Europe

Machine-accessible metadata file describing the reported data: 10.6084/m9.figshare.11294993

## Background & Summary

Tree rings are an important archive of individual life history variation with a large range of applications across both natural and social sciences. In their annual growth rings, trees chronologically record the effect of any factor – ranging from local to global scale – that directly or indirectly affects radial growth processes^1^. Typical factors influencing annual tree growth are for example growing season temperatures or soil water availability in cold and arid regions, respectively. Large datasets from tree-ring series sampled across wide geographical ranges have been used in a broad range of studies, e.g. to infer climate variability^2–4^, reconstruct variation in streamflow^5^, investigate processes affecting forest dynamics^6,7^, identify the origin of wood used in ancient buildings^8^, and date historical tools and instruments^9,10^.

The most important archive of tree-ring data worldwide is the International Tree-Ring Data Bank (ITRDB^11^). It has more than 4250 centrally-held datasets of 226 tree species from all continents, except Antarctica^12^. However, most of these tree-ring datasets were obtained following classical dendrochronological protocols, which usually aim to maximize the climatic signals recorded in the ring-width series by sampling climatically stressed and old populations^1^. Such a sampling design is convenient for climate reconstructions but can lead to bias in terms of climate sensitivity when using these datasets to elucidate ecological and evolutionary processes^13,14^. This is particularly relevant given that classic site selection criteria predominantly targeted extreme micro-site conditions (e.g., ridge or treeline locations), and selectively excluded measurements with weak common growth signal.

The increasing use of dendrochronological techniques in transdisciplinary studies^15^ is driving demand for improved ecological representativeness in the global tree-ring archives, in particular by adding new datasets from non-stressed populations, which help better representing the full environmental niche of the species^14^. One example is the recent combination of evolutionary biology and dendrochronology to assess signs of local adaptation in trees by linking phenotypes inferred from tree rings to genomic and environmental information^16–18^. The advantage that dendrochronology provides in this context is that the outcome of a wide variety of growth-related traits acting over the lifespan of an individual can be inferred from a single sample (wood core). Such kind of integrated phenotypes can then be investigated together with other datasets, although the association analyses should also take in consideration that some external processes such as disturbances or forest dynamics can affect tree growth and limit the potential genetic and climatic information encoded in the tree-ring series. Following this approach, the European project GenTree (http://www.gentree-h2020.eu) aims to provide the first comprehensive pan-European assessment of phenotypic and genomic variation within and among environmentally contrasted populations across multiple tree species. To this end, the GenTree consortium has collected a dataset of tree-core characteristics from 142 sites located across the geographical range of seven ecologically and economically important European tree species. Measurements include the widths of all annual rings dated to the exact calendar year of formation, and whole-core wood density (measured for 125 sites), as well as complementary information at tree level such as tree height and diameter at stem breast height (DBH).

Here, we present this pan-European dataset of tree-ring width series and other fitness-related traits that cover wide geographical ranges and contrasting habitats of the studied species. Despite the limitation given by an underrepresented selection of individuals at each site (only 25), the potential of this dataset goes far beyond the GenTree project goals and will also be of value for assessing and/or modelling forest properties under climate change scenarios.

## Methods

### Site selection

Sampling covered seven of the most ecologically and economically important tree species in Europe: silver birch (*Betula pendula* Roth), European beech (*Fagus sylvatica* L.), Norway spruce (*Picea abies* (L.) Karst), European black poplar (*Populus nigra* L.), maritime pine (*Pinus pinaster* Aiton), Scots pine (*Pinus sylvestris* L.), and sessile oak (*Quercus petraea* (Matt.) Liebl.). Sites were selected using the following criteria: i) natural populations with no clear signs of natural or anthropogenic disturbances, ii) within or near to a EUFORGEN Gene Conservation Unit (http://portal.eufgis.org/search/), iii) no infrastructure at close proximity (houses, roads, electric cables, larges pipes), iv) no extreme slope, and v) reasonably accessible. Each sampled tree was georeferenced using hand-held global positioning systems. The centroid of all tree individuals was used to estimate the geographical position of each site. Elevation was extracted for each site using the global multi-resolution terrain elevation data 2010 (GMTED2010)^19^.

**Fig. 1:**
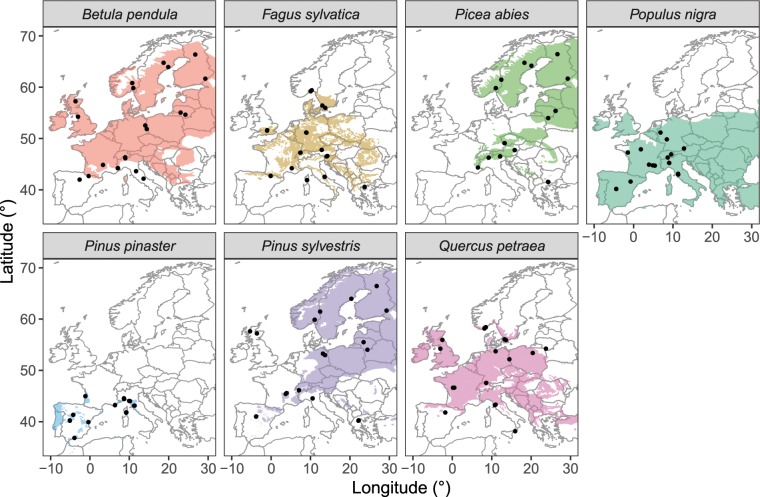
Natural distributions of the seven selected tree species and the geographical location of each study site from which tree-ring width measurements were obtained (142 sites in total). Distribution maps were obtained from EUFORGEN (www.euforgen.org).

**Fig. 2:**
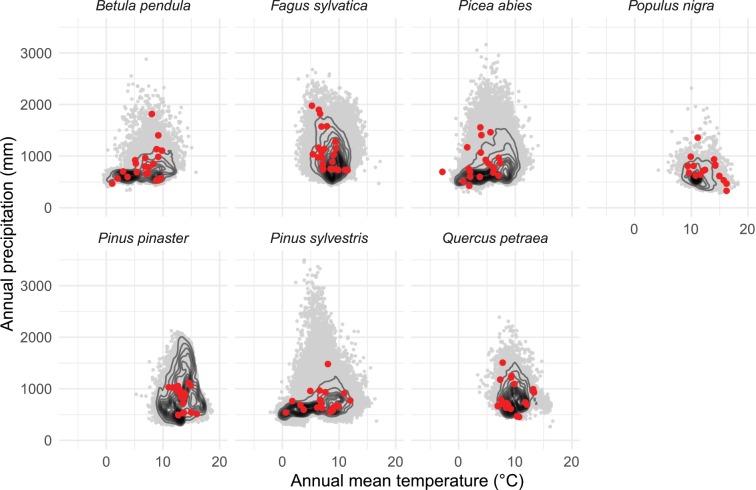
Climate-space diagrams for each species based on annual mean temperature and annual precipitation. Grey points represent species occurrences from across their total climate-space and red points show the climatic position of the selected study sites from which tree-ring measurements were obtained.

Sites were distributed across most of the geographical range of the species in Europe (Fig. 1). Climate-space diagrams were used to assess the relative climatic positions of each study site based on mean annual temperature and annual precipitation (Fig. 2). The geographical coordinates of tree occurrence in Europe were obtained from a reference publication^20^, and the corresponding climate data for the period 1979–2013 was extracted from CHELSA^21^ to plot full climate-space for each species, and overlaid the study sites on this distribution. The resulting plots show that the study sites are located in heterogeneous environmental conditions across a broad span of the climatic spaces occupied by the study species, covering contrasting habitats (Fig. 2).

### Sampling and laboratory protocols

Sampling took place from 2016 to 2018. At each study site, 25 dominant or co-dominant trees were selected. All trees were >25 m apart from each other to minimize the risk of sampling closely related individuals. Trees with visual symptoms of decay, infections, scars or abnormally low vigor were avoided. Each sampled tree was permanently labelled and one to three increment cores (depending on owner permission) were extracted at breast height (1.3 m) and perpendicular to the slope direction to avoid sampling reaction wood. Two cores were taken from one side of the stem and a third core from the opposite side. DBH was measured with a tape and height was estimated from ground to top of the tree using a clinometer.

The best core per tree, i.e. the core that was closest to the pith, without breakage or other obvious defects, was selected to conduct ring width measurements. Cores were air dried, mounted on wooden support beams, and then sanded with progressively finer sanding paper until wood cells were clearly visible under a binocular microscope. For silver birch and European black poplar, cores were surfaced along their cross-section using a core-microtome^22^ to obtain a clean cut plane surface facilitating the recognition of ring boundaries. Ring widths were measured to an accuracy of 0.01 mm using a binocular microscope connected to a LINTAB measuring device (Rinntech, Heidelberg, Germany). The exact year of formation was assigned to every annual ring through the cross-dating process^1,23^, by first visually cross-dating the tree-ring width series and then statistically verifying dating quality using the software CooRecorder (Cybis Elektronik & Data AB, Saltsjöbaden, Sweden). Missing rings, i.e. those that were absent within a series, were also actively detected and inserted into the series during the cross-dating process. Tree age at the coring height of 1.3 m was calculated as the length of the cross-dated tree-ring width series plus the estimated number of absent rings in the wood core towards the pith. The latter was estimated by fitting a template of concentric circles with known radii to the curve of the innermost rings and transforming the missing radius length into the number of absent rings. A summary of these parameters per population and species is reported in Online-only Table 1.

Whole-core wood density was determined on the second-best core (when available) and, in case the core was broken in several pieces, it was measured using the longest section. Wood volume was determined by the water-displacement method: the sample was immersed in a water-filled tray, which was placed on a balance. Weight of the displaced water was then converted to sample volume. Sample weight was measured on samples that had been dried in an oven at 102 °C for >2 hours (time required to obtain stable weights as reported by previous tests). Finally, wood density was obtained by dividing the sample weight by the sample volume.

The third core was kept as a reserve for any additional analyses. All original cores are stored at the Dendrosciences wood sample archives of the Swiss Federal Research Institute WSL in Birmensdorf (Switzerland) and can be made accessible upon request.

### Data records

The dataset is composed of three comma-separated files, and one metadata file, which are freely accessible at Figshare repository^24^. The first file (site.csv) contains site descriptions including site identifier, geographical coordinates, elevation, and the contact details of the site coordinator. The second file (tree.csv) provides information at tree level, namely geographical coordinates, length of the ring width series, distance to the pith, estimated tree age, stem DBH, tree height, an assessment of dating confidence, and wood core density. The third file (trw_long_format.csv) contains annually-resolved tree ring width measurements of all trees included in the study (3600 trees in total). Missing values in the first two files (site.csv, and tree.csv) are denoted by NA. Missing ring measurements, defined as those actively detected during the cross-dating process, are denoted by 0 in the trw.csv file. The metadata file contains all definitions and unit for each variable. Additionally, rwl files of all sites containing the individual tree-ring width series (exact information than the trw_long_format.csv) are also included.

Both metadata and data files can also be accessed on the GnpIS information system at the following Gnpis Repository^25^. There, data will be updated as new data on additional GenTree species (*Abies alba* Mill., *Pinus cembra* L., *Pinus halepensis* Mill., *Pinus nigra* Arn., and *Taxus baccata* L.) are provided by partners.

### Technical validation

Multiple steps were taken to ensure the technical quality of the measurements. The correct dating and quality of cross-dating was checked statistically with the software COFECHA^26^. It correlates each individual ring width series with the overall mean site series (after removing the series being tested). This analysis identifies mismatches and mistakes in the ring width measurements. The mean intercorrelation between raw individual series from the same site (Rbt.raw) was calculated and showed good within-site agreement (Online-only Table 1, Fig. S1). The expressed population signal (EPS), a measure of how well the mean series represents the common variability of the entire population if it were infinitely replicated, was also used to check the data quality. Low values of EPS usually indicate that the mean site series is influenced by individual processes rather than a consistent common signal. EPS values were calculated on the high-frequency domain (year-to-year variability) of the measured series. To do so, the low-frequency variability (decadal) was removed from the raw tree-ring series by applying a 32-year spline to each individual series. EPS was calculated in two different ways. To have an overview of the common variability shared by all the trees of a given site, EPS was calculated taking in consideration the common time period. In this case, most of the site chronologies (81%) presented an EPS above 0.85 (accepted threshold for signal strength in dendrochronological studies^27^) and only 19% showed an EPS lower than 0.85 (Online-only Table 1, Fig. S1). Due to the heterogenous age of the trees included in each site, we also calculated the EPS of the last 25 years but aiming at optimizing the maximum pairwise overlap. Similar percentages of sites presenting EPS above and below 0.85 were obtained (80% and 20%, respectively), but some of the sites that previously showed extremely low EPS values improved their EPS to reasonable values when assessing the maximum pairwise overlap. In general, low EPS values can be caused by a variety of factors such as short series length (not only old trees were selected), or low suitability for dendrochronological studies of some tree species such as silver birch and European black poplar. The sampling design did not specifically aim at selecting climatically limited populations, and consequently, the common signal of some sites might not be as strong as usually expected in dendrochronological studies, resulting in low EPS values. For this reason, and to complement the statistical assessment, the cross-dating confidence level of each dated ring series was classified (A = high confidence, B = possible doubts, C = very questionable). “A” letter was assigned to tree cores that were easily cross-dated and were well correlated with the rest of samples, “B” letter was assigned to cores with intermediate agreement with the rest of samples and/or showing small cracks in the wood, and “C” letter was assigned to tree cores that presented relatively low agreement with the rest of samples. The mean intercorrelation between detrended individual series from the same site was also calculated (Rbt.det, Online-only Table 1), which corroborated the generally good agreement among series in the high-frequency domains.

**Fig. 3:**
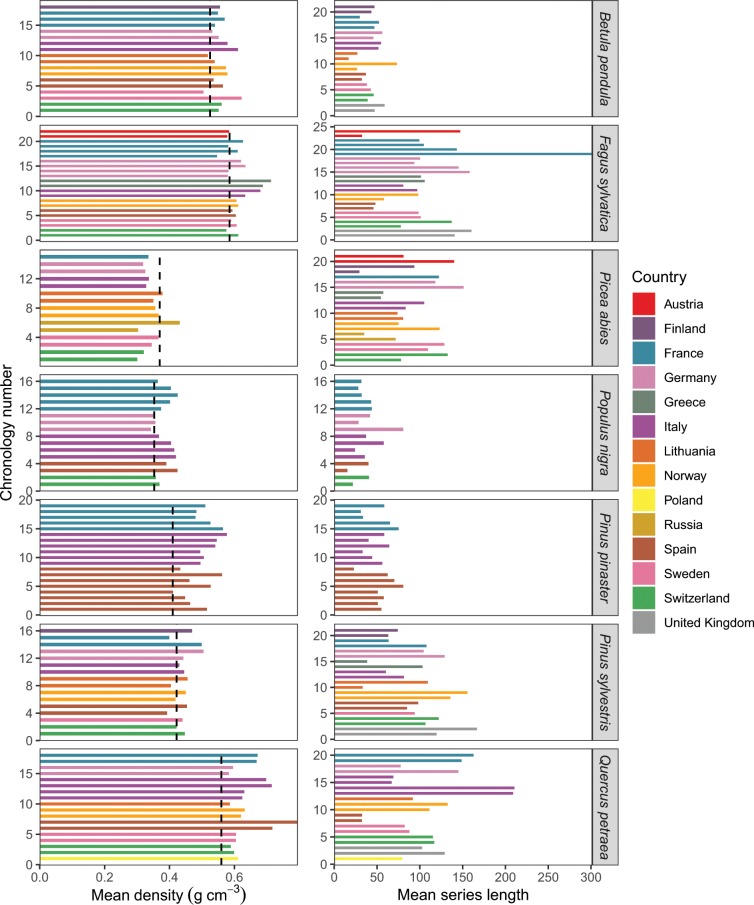
Mean density and mean series length for each tree species and site. Vertical dashed black lines indicated the reference mean wood density values.

The average wood density per species was compared to those from a reference dataset^28,29^ (Fig. 3). As in this dataset, Norway spruce, black poplar, Scots pine, and maritime pine displayed lower mean density values than the ones obtained for silver birch, European beech and sessile oak (Online-only Table 1, Figs. 3 and S2).

### Supplementary information

Supplementary File

## Online-only Table

**Online-only Table 1:** Descriptors and statistics of the ring width series for the 142 measured sites.

SiteID	Species	Country	N tree	MSL	Mage (year)	MRW (mm)	Rbt raw	Rbt det	Common period	EPS	EPS (last 25y)	Mdensity (g cm^−3^)
FIBP19	*Betula pendula*	Finland	25	47	48.16	1.93	0.27	0.3	2015-1990	0.923	0.924	NA
FIBP20	*Betula pendula*	Finland	25	43.4	45.56	2.07	0.31	0.25	2015-1998	0.858	0.900	0.556
FRBP03	*Betula pendula*	France	25	29.8	31.25	3.08	0.28	0.23	2013-2004	0.923	0.892	0.549
FRBP04	*Betula pendula*	France	25	52.32	55.04	2.49	0.49	0.24	2015-1985	0.895	0.898	0.57
FRBP21	*Betula pendula*	France	25	46.88	49.08	1.87	0.11	0.09	2007-2005	0.510	0.741	0.54
DEBP09	*Betula pendula*	Germany	25	56.16	61.23	2.28	0.39	0.14	2010-1991	0.809	0.804	0.532
DEBP10	*Betula pendula*	Germany	26	45.77	47.76	1.6	0.56	0.36	2006-1976	0.942	0.932	0.552
ITBP07	*Betula pendula*	Italy	25	54.68	56.13	2.01	0.46	0.05	2012-1979	0.497	0.402	0.579
ITBP08	*Betula pendula*	Italy	25	51.64	54.72	1.36	0.18	0.16	2013-1990	0.741	0.840	0.611
LTBP11	*Betula pendula*	Lithuania	25	27	25.52	4.06	0.4	0.32	2016-2000	0.911	0.831	0.518
LTBP12	*Betula pendula*	Lithuania	25	16.76	17.74	5.15	0.57	0.48	2016-2004	0.953	0.960	0.539
NOBP15	*Betula pendula*	Norway	25	73.24	70.59	1.43	0.27	0.22	2016-1977	0.893	0.889	0.574
NOBP16	*Betula pendula*	Norway	25	26.68	27.24	3.87	0.54	0.32	2016-1996	0.922	0.919	0.578
ESBP01	*Betula pendula*	Spain	25	37	40.16	3.67	0.33	0.26	2012-1994	0.921	0.907	0.536
ESBP02	*Betula pendula*	Spain	25	32.16	34.92	2.87	0.25	0.2	2015-2004	0.845	0.893	0.565
SEBP17	*Betula pendula*	Sweden	25	38.12	40.52	2.73	0.36	0.32	2015-1999	0.930	0.935	0.505
SEBP18	*Betula pendula*	Sweden	25	42.84	43.48	2.56	0.35	0.25	2015-1993	0.887	0.882	0.622
CHBP05	*Betula pendula*	Switzerland	25	46.04	51.09	2.9	0.37	0.19	2015-1992	0.842	0.837	0.561
CHBP06	*Betula pendula*	Switzerland	25	39.04	41.42	2.78	0.18	0.08	2011-1992	0.691	0.779	0.551
GBBP13	*Betula pendula*	United Kingdom	24	58.83	62.41	1.82	0.29	0.15	2010-1986	0.827	0.828	NA
GBBP14	*Betula pendula*	United Kingdom	24	47.21	49.54	2.23	0.25	0.12	2016-2007	0.804	0.815	NA
Mean				43.46	45.41	2.61	0.35	0.23		0.834	0.848	0.557
ATFS13	*Fagus sylvatica*	Austria	22	147.18	150.81	0.61	0.25	0.16	2015-1960	0.871	0.863	0.584
ATFS14	*Fagus sylvatica*	Austria	25	32.56	33.8	3.11	0.09	0.09	2015-2001	0.760	0.616	0.578
FRFS03	*Fagus sylvatica*	France	25	99.44	103.2	1.17	0.44	0.52	2015-1946	0.970	0.976	0.627
FRFS04	*Fagus sylvatica*	France	25	104.6	105.6	1.03	0.26	0.31	2015-1937	0.913	0.944	0.581
FRFS05	*Fagus sylvatica*	France	25	143.2	148.58	1.7	0.2	0.33	2015-1944	0.953	0.959	0.61
FRFS06	*Fagus sylvatica*	France	25	301.44	369.29	1.01	0.32	0.32	2015-1963	0.951	0.940	0.546
DEFS15	*Fagus sylvatica*	Germany	25	100.36	102.75	1.59	0.27	0.31	2016-1931	0.911	0.884	0.62
DEFS16	*Fagus sylvatica*	Germany	26	93.62	95.38	1.61	0.19	0.32	2015-1938	0.932	0.875	0.634
DEFS17	*Fagus sylvatica*	Germany	25	145.24	170.42	0.89	0.38	0.5	2015-1961	0.968	0.968	0.582
DEFS18	*Fagus sylvatica*	Germany	25	158.24	168.48	1.6	0.46	0.51	2015-1944	0.962	0.958	0.58
GRFS09	*Fagus sylvatica*	Greece	25	101.32	105.2	1.64	0.22	0.3	2008-1965	0.874	0.898	0.713
GRFS10	*Fagus sylvatica*	Greece	25	105.68	110.31	1.48	0.13	0.18	2016-1960	0.877	0.877	0.688
ITFS07	*Fagus sylvatica*	Italy	25	80.64	82.28	2.2	0.21	0.14	2008-1968	0.823	0.853	0.68
ITFS08	*Fagus sylvatica*	Italy	25	97	99.64	1.9	0.3	0.33	2016-1961	0.927	0.945	0.633
NOFS21	*Fagus sylvatica*	Norway	25	98.08	99.38	1.15	0.26	0.36	2015-1971	0.956	0.947	0.606
NOFS22	*Fagus sylvatica*	Norway	25	58.2	59.2	1.94	0.26	0.37	2015-2000	0.956	0.963	0.612
ESFS01	*Fagus sylvatica*	Spain	25	48.08	52	2.6	0.12	0.2	2015-1993	0.858	0.842	0.594
ESFS02	*Fagus sylvatica*	Spain	25	45.88	48.28	2.42	0.16	0.19	2015-1995	0.859	0.885	0.604
SEFS23	*Fagus sylvatica*	Sweden	25	98.44	101.32	2.01	0.25	0.34	2015-1974	0.945	0.943	0.59
SEFS24	*Fagus sylvatica*	Sweden	25	101.16	103.27	1.88	0.18	0.17	2003-1955	0.858	0.898	0.607
CHFS11	*Fagus sylvatica*	Switzerland	25	137.24	144.5	0.94	0.29	0.34	2015-1959	0.921	0.929	0.575
CHFS12	*Fagus sylvatica*	Switzerland	25	77.84	82.64	2.08	0.31	0.21	2015-1963	0.882	0.905	0.612
GBFS19	*Fagus sylvatica*	United Kingdom	30	160.37	166.57	1.61	0.2	0.29	2017-1915	0.948	0.968	NA
GBFS20	*Fagus sylvatica*	United Kingdom	27	140.67	144.74	1.63	0.19	0.15	2017-1955	0.886	0.922	NA
Mean				111.52	118.65	1.66	0.25	0.29		0.906	0.907	0.612
ATPA05	*Picea abies*	Austria	25	80.92	85	1.6	0.12	0.15	2015-1988	0.893	0.892	NA
ATPA06	*Picea abies*	Austria	25	140.08	151.89	0.68	0.4	0.21	2015-1955	0.764	0.798	NA
FIPA17	*Picea abies*	Finland	25	93.52	94.96	1.2	0.28	0.37	2015-1991	0.917	0.917	NA
FIPA18	*Picea abies*	Finland	25	29.36	30.24	2.26	0.27	0.22	2015-2002	0.860	0.878	NA
FRPA21	*Picea abies*	France	25	122.28	124.46	1.96	0.13	0.2	2015-1978	0.900	0.899	0.335
DEPA09	*Picea abies*	Germany	25	118.08	122.06	2.44	0.22	0.17	2008-1984	0.868	0.872	0.319
DEPA10	*Picea abies*	Germany	25	151.04	159.42	1.51	0.24	0.3	2015-1986	0.935	0.935	0.325
GRPA07	*Picea abies*	Greece	24	57.25	61.24	3.09	0.16	0.19	2016-1998	0.814	0.823	NA
GRPA08	*Picea abies*	Greece	25	54.44	57.21	3.79	0.12	0.17	2016-1998	0.886	0.841	NA
ITPA03	*Picea abies*	Italy	25	105.12	108.7	1.76	0.1	0.23	2013-1967	0.907	0.902	0.336
ITPA04	*Picea abies*	Italy	25	83.44	85.08	1.67	0.19	0.28	2014-1981	0.939	0.950	0.328
LTPA11	*Picea abies*	Lithuania	25	73.96	85.18	2.74	0.25	0.29	2015-1981	0.916	0.934	0.378
LTPA12	*Picea abies*	Lithuania	24	80.54	84.81	2.13	0.17	0.25	2015-1961	0.885	0.870	0.35
NOPA13	*Picea abies*	Norway	25	75.16	78.28	1.93	0.21	0.35	2015-1988	0.948	0.952	0.356
NOPA14	*Picea abies*	Norway	25	123.04	125.16	0.93	0.23	0.37	2015-1942	0.945	0.936	0.367
RUPA19	*Picea abies*	Russia	23	35	39.32	1.45	0.21	0.22	2012-2006	0.860	0.909	0.432
RUPA20	*Picea abies*	Russia	25	71.72	83.44	3.01	0.32	0.3	2015-1982	0.928	0.924	0.303
SEPA15	*Picea abies*	Sweden	25	128.72	138.91	0.91	0.25	0.36	2010-1965	0.942	0.944	0.366
SEPA16	*Picea abies*	Sweden	25	109.56	111.52	1.51	0.36	0.44	2015-1944	0.961	0.971	0.345
CHPA01	*Picea abies*	Switzerland	25	132.52	140.17	1.95	0.28	0.24	2008-1972	0.940	0.958	0.321
CHPA02	*Picea abies*	Switzerland	25	78.08	75.75	3.15	0.17	0.17	2015-1989	0.860	0.867	0.301
Mean				92.56	97.28	1.98	0.22	0.26		0.898	0.903	0.344
FRPP09	*Pinus pinaster*	France	30	58.5	59.62	2.51	0.66	0.45	2016-1965	0.956	0.971	0.511
FRPP11	*Pinus pinaster*	France	25	31.16	36.2	3.02	0.33	0.2	2016-1999	0.852	0.879	0.483
FRPP12	*Pinus pinaster*	France	25	33.64	39.44	3.4	0.53	0.36	2013-1994	0.940	0.942	0.48
FRPP13	*Pinus pinaster*	France	25	65.16	66.88	3.15	0.53	0.4	2016-1961	0.942	0.933	0.526
FRPP14	*Pinus pinaster*	France	25	75.2	79.4	4.11	0.44	0.23	2016-1983	0.844	0.833	0.565
ITPP15	*Pinus pinaster*	Italy	25	58.4	62.08	3	0.48	0.23	2012-1985	0.870	0.853	0.577
ITPP16	*Pinus pinaster*	Italy	25	40.16	42.46	4.56	0.22	0.11	2013-2009	0.267	0.879	0.546
ITPP17	*Pinus pinaster*	Italy	25	64.24	67.8	2	0.46	0.39	2008-1980	0.958	0.952	0.541
ITPP18	*Pinus pinaster*	Italy	25	33.08	36.44	2.83	0.47	0.36	2015-2003	0.917	0.926	0.495
ITPP19	*Pinus pinaster*	Italy	25	44.36	45.48	2.24	0.39	0.1	2013-2005	0.751	0.761	0.506
ITPP20	*Pinus pinaster*	Italy	25	56.28	57.76	1.53	0.6	0.25	2015-1976	0.908	0.911	0.495
ESPP01	*Pinus pinaster*	Spain	25	22.96	24.04	6.65	0.12	0.13	2015-2010	0.877	0.826	0.433
ESPP02	*Pinus pinaster*	Spain	25	62.44	55.14	3.01	0.14	0.21	2015-2005	0.936	0.906	0.562
ESPP03	*Pinus pinaster*	Spain	25	70.28	72.72	2.57	0.46	0.44	2015-1966	0.952	0.947	0.462
ESPP04	*Pinus pinaster*	Spain	25	80.6	82.67	2.14	0.39	0.34	2013-1979	0.931	0.931	0.527
ESPP05	*Pinus pinaster*	Spain	25	50.8	52.44	2.34	0.67	0.54	2001-1982	0.964	0.977	0.411
ESPP06	*Pinus pinaster*	Spain	25	57.92	61.26	3.11	0.32	0.17	2015-1982	0.851	0.862	0.448
ESPP07	*Pinus pinaster*	Spain	25	50.96	52.28	2.49	0.52	0.57	2013-2002	0.966	0.985	0.464
ESPP08	*Pinus pinaster*	Spain	25	55.08	56.79	2.13	0.44	0.53	2014-1996	0.977	0.973	0.515
Mean				53.22	55.31	2.99	0.42	0.32		0.876	0.908	0.502
FIPS18	*Pinus sylvestris*	Finland	25	74.16	75.6	1.1	0.58	0.32	2015-1962	0.922	0.918	NA
FIPS19	*Pinus sylvestris*	Finland	25	63.16	65.88	1.96	0.45	0.42	2015-1980	0.954	0.955	0.469
FRPS03	*Pinus sylvestris*	France	26	63.5	64.36	2.61	0.56	0.36	2015-1980	0.937	0.950	0.399
FRPS04	*Pinus sylvestris*	France	25	107.76	111.83	1.53	0.44	0.31	2015-1918	0.923	0.928	0.499
DEPS11	*Pinus sylvestris*	Germany	26	104.58	108	1.42	0.51	0.39	2013-1922	0.945	0.934	0.505
DEPS12	*Pinus sylvestris*	Germany	26	129	126.7	1.57	0.18	0.26	2001-1963	0.883	0.878	0.443
GRPS09	*Pinus sylvestris*	Greece	25	38.48	39.95	4.54	0.46	0.2	2013-2001	0.854	0.874	NA
GRPS10	*Pinus sylvestris*	Greece	25	103.08	114	2.55	0.45	0.2	2009-1954	0.865	0.854	NA
ITPS07	*Pinus sylvestris*	Italy	25	60.32	62.56	1.85	0.51	0.32	2015-1993	0.913	0.914	0.43
ITPS08	*Pinus sylvestris*	Italy	25	81.48	83.16	1.22	0.38	0.23	2015-1973	0.899	0.920	0.445
LTPS20	*Pinus sylvestris*	Lithuania	25	109.36	110.08	1.47	0.3	0.39	2014-1958	0.940	0.955	0.456
LTPS21	*Pinus sylvestris*	Lithuania	21	33.24	34.45	1.05	0.33	0.16	2006-2001	0.672	0.695	0.404
NOPS15	*Pinus sylvestris*	Norway	25	155.72	157.96	0.85	0.2	0.29	2015-1917	0.923	0.877	0.45
NOPS16	*Pinus sylvestris*	Norway	25	135.72	137.48	0.99	0.27	0.31	2015-1946	0.936	0.941	0.419
ESPS01	*Pinus sylvestris*	Spain	25	98.08	100.09	1.79	0.66	0.39	2014-1935	0.939	0.954	0.454
ESPS02	*Pinus sylvestris*	Spain	25	85	86.52	1.82	0.66	0.43	2013-1950	0.955	0.948	0.392
SEPS17	*Pinus sylvestris*	Sweden	25	94	95.92	1.35	0.56	0.3	2007-1958	0.926	0.928	0.44
CHPS05	*Pinus sylvestris*	Switzerland	25	122.08	123.04	1.45	0.26	0.27	2015-1981	0.900	0.861	0.422
CHPS06	*Pinus sylvestris*	Switzerland	25	106.6	109.88	1.58	0.41	0.35	1983-1979	0.917	0.907	0.447
GBPS13	*Pinus sylvestris*	United Kingdom	28	166.75	176	1.52	0.21	0.23	2018-1988	0.905	0.886	NA
GBPS14	*Pinus sylvestris*	United Kingdom	25	119.64	125.13	2.29	0.46	0.16	2011-1996	0.893	0.898	NA
Mean				97.7	100.41	1.74	0.42	0.30		0.905	0.904	0.442
FRPO04	*Populus nigra*	France	29	31.72	34.38	8.46	0.35	0.18	2016-2004	0.910	0.868	0.364
FRPO05	*Populus nigra*	France	25	28.08	26.35	8.47	0.17	0.16	2016-2011	0.274	0.791	0.404
FRPO06	*Populus nigra*	France	28	32.07	37.48	5.16	0.32	0.13	2013-2004	0.529	0.888	0.425
FRPO07	*Populus nigra*	France	29	43.1	46.41	3.6	0.12	0.05	2016-2002	0.676	0.544	0.402
FRPO20	*Populus nigra*	France	29	43.9	31.69	6.02	0.36	0.12	2016-1995	0.773	0.760	0.374
DEPO08	*Populus nigra*	Germany	25	41.84	46.47	6.25	0.43	0.26	2016-1994	0.897	0.891	0.353
DEPO09	*Populus nigra*	Germany	25	28.44	30.2	4.34	0.59	0.44	2016-1995	0.958	0.768	0.357
DEPO10	*Populus nigra*	Germany	25	80.64	86.13	3.77	0.23	0.16	2001-1987	0.834	0.953	0.343
ITPO14	*Populus nigra*	Italy	25	37.16	35.44	9.06	0.22	0.15	2015-2011	0.792	0.848	0.367
ITPO15	*Populus nigra*	Italy	25	57.88	66.5	4.49	0.18	0.17	2010-2000	0.865	0.891	0.405
ITPO16	*Populus nigra*	Italy	25	24.24	27.65	5.61	0.42	0.44	2015-2007	0.959	0.807	0.415
ITPO17	*Populus nigra*	Italy	25	35.68	39.95	4.84	0.39	0.13	1999-1997	0.000	0.967	0.42
ESPO01	*Populus nigra*	Spain	21	40.05	41.8	5	0.18	0.19	2015-2006	0.865	0.846	0.39
ESPO02	*Populus nigra*	Spain	25	15.44	18.29	8.57	0.22	0.23	2016-2013	0.933	0.916	0.424
CHPO12	*Populus nigra*	Switzerland	32	40.59	40.9	4.75	0.36	0.35	2015-2006	0.947	0.945	0.358
CHPO13	*Populus nigra*	Switzerland	27	21.78	23.96	5.62	0.13	0.14	2013-2006	0.745	0.780	0.369
Mean				37.66	39.6	5.87	0.29	0.21		0.747	0.841	0.386
FRQP03	*Quercus petraea*	France	25	162.8	189.38	1.66	0.25	0.36	1965-1927	0.951	0.880	0.671
FRQP04	*Quercus petraea*	France	25	148.72	155.95	1.22	0.28	0.35	2015-1928	0.916	0.969	0.668
DEQP17	*Quercus petraea*	Germany	26	77.54	79.12	2.03	0.3	0.36	2015-1945	0.930	0.914	0.596
DEQP18	*Quercus petraea*	Germany	27	145.04	147.12	1.19	0.44	0.51	2016-1896	0.961	0.942	0.583
ITQP07	*Quercus petraea*	Italy	25	69.12	70.88	1.84	0.59	0.27	2016-1966	0.920	0.909	0.698
ITQP08	*Quercus petraea*	Italy	25	67.12	70.42	1.05	0.62	0.21	2011-1981	0.793	0.842	0.715
ITQP09	*Quercus petraea*	Italy	25	210.48	257.44	0.75	0.12	0.11	2016-1958	0.870	0.857	0.631
ITQP10	*Quercus petraea*	Italy	25	208.92	272	0.8	0.18	0.14	2014-1972	0.874	0.864	0.624
LTQP20	*Quercus petraea*	Lithuania	25	91.8	104	1.35	0.29	0.35	2015-1968	0.934	0.940	0.586
NOQP13	*Quercus petraea*	Norway	25	132.56	138.18	1.19	0.25	0.43	2015-1975	0.957	0.946	0.632
NOQP14	*Quercus petraea*	Norway	25	111.24	112.72	0.94	0.36	0.51	2015-1968	0.973	0.970	0.62
POQP19	*Quercus petraea*	Poland	25	79.52	79.25	1.56	0.22	0.32	1994-1986	0.924	0.933	0.612
ESQP01	*Quercus petraea*	Spain	25	32.56	34.36	2.51	0.38	0.26	2015-1997	0.918	0.914	0.795
ESQP02	*Quercus petraea*	Spain	25	32.36	33.32	2.43	0.34	0.31	2015-1998	0.935	0.939	0.717
SEQP15	*Quercus petraea*	Sweden	25	82.36	84.16	1.84	0.3	0.43	2015-1945	0.950	0.945	0.605
SEQP16	*Quercus petraea*	Sweden	25	87.72	89.72	1.76	0.26	0.37	2014-1965	0.945	0.954	0.605
CHQP05	*Quercus petraea*	Switzerland	25	115.24	121.63	1.29	0.27	0.38	2015-1968	0.914	0.914	0.589
CHQP06	*Quercus petraea*	Switzerland	26	116.88	120.88	1.51	0.21	0.39	2015-1942	0.948	0.893	0.599
GBQP11	*Quercus petraea*	United Kingdom	21	102.67	101.92	1.94	0.45	0.37	2016-1961	0.909	0.886	NA
GBQP12	*Quercus petraea*	United Kingdom	25	129	127	1.77	0.38	0.32	2016-1999	0.885	0.901	NA
Mean				110.18	119.47	1.53	0.33	0.34		0.920	0.916	0.641

Ntree, number of trees; MSL, mean series length; Mage, mean age; MRW, mean ring width; Rbt.raw, mean intercorrelation among raw ring-width series; Rbt.det, mean intercorrelation among detrended ring width series; Common period, time period in common for all the trees from the same site; EPS, expressed population signal for the common period, EPS (last 25 y), expressed population signal calculated for the last 25 years for the maximum pairwise overlap; Mdensity; mean whole-core wood density. Note that EPS and Rbt.det have been calculated after applying a 32-year spline to raw series.
