# Author Correction: The GenTree Dendroecological Collection, tree-ring and wood density data from seven tree species across Europe

**DOI:** 10.1038/s41597-020-0447-1

**Published:** 2020-04-02

**Authors:** Elisabet Martínez-Sancho, Lenka Slámová, Sandro Morganti, Claudio Grefen, Barbara Carvalho, Benjamin Dauphin, Christian Rellstab, Felix Gugerli, Lars Opgenoorth, Katrin Heer, Florian Knutzen, Georg von Arx, Fernando Valladares, Stephen Cavers, Bruno Fady, Ricardo Alía, Filippos Aravanopoulos, Camilla Avanzi, Francesca Bagnoli, Evangelos Barbas, Catherine Bastien, Raquel Benavides, Frédéric Bernier, Guillaume Bodineau, Cristina C. Bastias, Jean-Paul Charpentier, José M. Climent, Marianne Corréard, Florence Courdier, Darius Danusevicius, Anna-Maria Farsakoglou, José M. García del Barrio, Olivier Gilg, Santiago C. González-Martínez, Alan Gray, Christoph Hartleitner, Agathe Hurel, Arnaud Jouineau, Katri Kärkkäinen, Sonja T. Kujala, Mariaceleste Labriola, Martin Lascoux, Marlène Lefebvre, Vincent Lejeune, Grégoire Le-Provost, Mirko Liesebach, Ermioni Malliarou, Nicolas Mariotte, Silvia Matesanz, Célia Michotey, Pascal Milesi, Tor Myking, Eduardo Notivol, Birte Pakull, Andrea Piotti, Christophe Plomion, Mehdi Pringarbe, Tanja Pyhäjärvi, Annie Raffin, José A. Ramírez-Valiente, Kurt Ramskogler, Juan J. Robledo-Arnuncio, Outi Savolainen, Silvio Schueler, Vladimir Semerikov, Ilaria Spanu, Jean Thévenet, Mari Mette Tollefsrud, Norbert Turion, Dominique Veisse, Giovanni Giuseppe Vendramin, Marc Villar, Johan Westin, Patrick Fonti

**Affiliations:** 1 Swiss Federal Institute for Forest Snow and Landscape Research WSL, Zürcherstrasse 111, 8903 Birmensdorf, Switzerland; 2 Philipps-Universität Marburg, Department of Biology, Karl-von-Frisch-Strasse 8, 35043 Marburg, Germany; 3 Department of Biogeography and Global Change, National Museum of Natural Sciences MNCN-CSIC, Serrano 115 dpdo, 28006 Madrid, Spain; 4 Bavarian Office for Forest Seeding and Planting – ASP, Forstamtsplatz 1, 83317 Teisendorf, Germany; 5 Centre for Ecology and Hydrology NERC, EH26 0QB Edinburgh, United Kingdom; 6 Institut National de la Recherche Agronomique (INRA), Domaine Saint Paul, Site Agroparc, 84914 Avignon, France; 7 Instituto Nacional de Investigación y Tecnología Agraria y Alimentaria - Centro de Investigación Forestal (INIA-CIFOR), Ctra. de la Coruña km 7.5, 28040 Madrid, Spain; 8 Laboratory of Forest Genetics and Tree Breeding, School of Forestry and Natural Environment, Aristotle University of Thessaloniki, 54124 Thessaloniki, Greece; 9 Institute of Biosciences and BioResources, National Research Council (CNR), via Madonna del Piano 10, 50019 Sesto Fiorentino, Italy; 10 Institut National de la Recherche Agronomique (INRA), Avenue de la Pomme de pin 2163, 45075 Orléans, France; 11 Institut National de la Recherche Agronomique (INRA), route d’Arcachon 69, 33610 Cestas, France; 12 Vytautas Magnus University, Studentu Street 11, 53361 Akademija, Lithuania; 13 LIECO GmbH & Co KG, Forstgarten 1, 8775 Kalwang, Austria; 14 Natural Resources Institute Finland, University of Oulu, Paavo Havaksen tie 3, 90014 Oulu, Finland; 15 Department of Ecology & Genetics, EBC, Uppsala University, Norbyvägen 18D, 75236 Uppsala, Sweden; 16 Thünen Institute of Forest Genetics, Sieker Landstr. 2, 22927 Grosshansdorf, Germany; 17 Área de Biodiversidad y Conservación, Universidad Rey Juan Carlos, Calle Tulipán s/n, 28933 Móstoles, Spain; 18 Université Paris-Saclay, INRAE, URGI, 78026 Versailles, France; 19 Department of Ecology and Genetics, Evolutionary Biology Center, Science for Life Laboratory, Uppsala University, Norbyvägen, 18 D, 752 36 Uppsala, Sweden; 20 Division of Forestry and Forest Resources, Norwegian Institute of Bioeconomy Research (NIBIO), P.O. Box 115, 1431 Ås, Norway; 21 Centro de Investigación y Tecnología Agroalimentaria de Aragón - Unidad de Recursos Forestales (CITA), Avda. Montañana 930, 50059 Zaragoza, Spain; 22 University of Oulu, Pentti Kaiteran katu 1, 90014 Oulu, Finland; 23 Austrian Research Centre for Forests (BFW), Seckendorff-Gudent-Weg 8, 1131 Wien, Austria; 24 Institute of Plant and Animal Ecology, Ural branch of RAS, 8 Marta St. 202, 620144 Ekaterinburg, Russia; 25 Skogforsk, Tomterna 1, 91821 Sävar, Sweden

Correction to: *Scientific Data* 10.1038/s41597-019-0340-y, published online 02 January 2020

The following authors were omitted from the original version of this Data Descriptor: Grégoire Le-Provost, Célia Michotey, Pascal Milesi and Christophe Plomion. The author list and affiliations have been amended to address these omissions.

In addition, a number of missing names have been added to the Acknowledgements section and an initial has been corrected in the Author Contributions section.

Both the HTML and PDF versions have been updated to reflect these changes.

