# Supplementary material for: The GenTree Dendroecological Collection, tree-ring and wood density data from seven tree species across Europe
Source: Sci Data. 2020 Jan 2;7:1. doi: 10.1038/s41597-019-0340-y (PMC6940356; doi:10.1038/s41597-019-0340-y)
Supplement: Supplementary file 1 — Supplementary File [file 41597_2019_340_MOESM1_ESM.docx]

**The GenTree Dendroecological Collection, tree-ring and wood density data from seven tree species across Europe**

Elisabet Martínez-Sancho^1*^, Lenka Slámová^1^, Sandro Morganti^1^, Claudio Grefen^2^, Barbara Carvalho^3^, Benjamin Dauphin^1^, Christian Rellstab^1^, Felix Gugerli^1^, Lars Opgenoorth^2^, Katrin Heer^2^, Florian Knutzen^4^, Georg von Arx^1^, Fernando Valladares^3^, Stephen Cavers^5^, Bruno Fady^6^, Ricardo Alía^7^, Filippos Aravanopoulos^8^, Camilla Avanzi^9^, Francesca Bagnoli^9^, Evangelos Barbas^8^, Catherine Bastien^10^, Raquel Benavides^3^, Frédéric Bernier^11^, Guillaume Bodineau^10^, Cristina C. Bastias^3^, Jean-Paul Charpentier^10^, José M. Climent^7^, Marianne Corréard^6^, Florence Courdier^6^, Darius Danusevicius^12^, Anna-Maria Farsakoglou^8^, José M. García del Barrio^7^, Olivier Gilg^6^, Santiago C. González-Martínez^11^, Alan Gray^5^, Christoph Hartleitner^13^, Agathe Hurel^11^, Arnaud Jouineau^6^, Katri Kärkkäinen^14^, Sonja Kujala^14^, Mariaceleste Labriola^9^, Martin Lascoux^15^, Marlène Lefebvre^10^, Vincent Lejeune^10^, Mirko Liesebach^16^, Ermioni Malliarou^8^, Nicolas Mariotte^6^, Silvia Matesanz^17^, Tor Myking^18^, Eduardo Notivol^19^, Birte Pakull^16^, Andrea Piotti^9^, Mehdi Pringarbe^6^, Tanja Pyhäjärvi^20^, Annie Raffin^11^, José A. Ramírez-Valiente^7^, Kurt Ramskogler^13^, Juan J. Robledo-Arnuncio^7^, Outi Savolainen^20^, Silvio Schueler^21^, Vladimir Semerikov^22^, Ilaria Spanu^9^, Jean Thévenet^6^, Mari Mette Tollefsrud^17^, Norbert Turion^6^, Dominique Veisse^10^, Giovanni Giuseppe Vendramin^9^, Marc Villar^10^, Johan Westin^23^ and Patrick Fonti^1^

**Supplementary information**


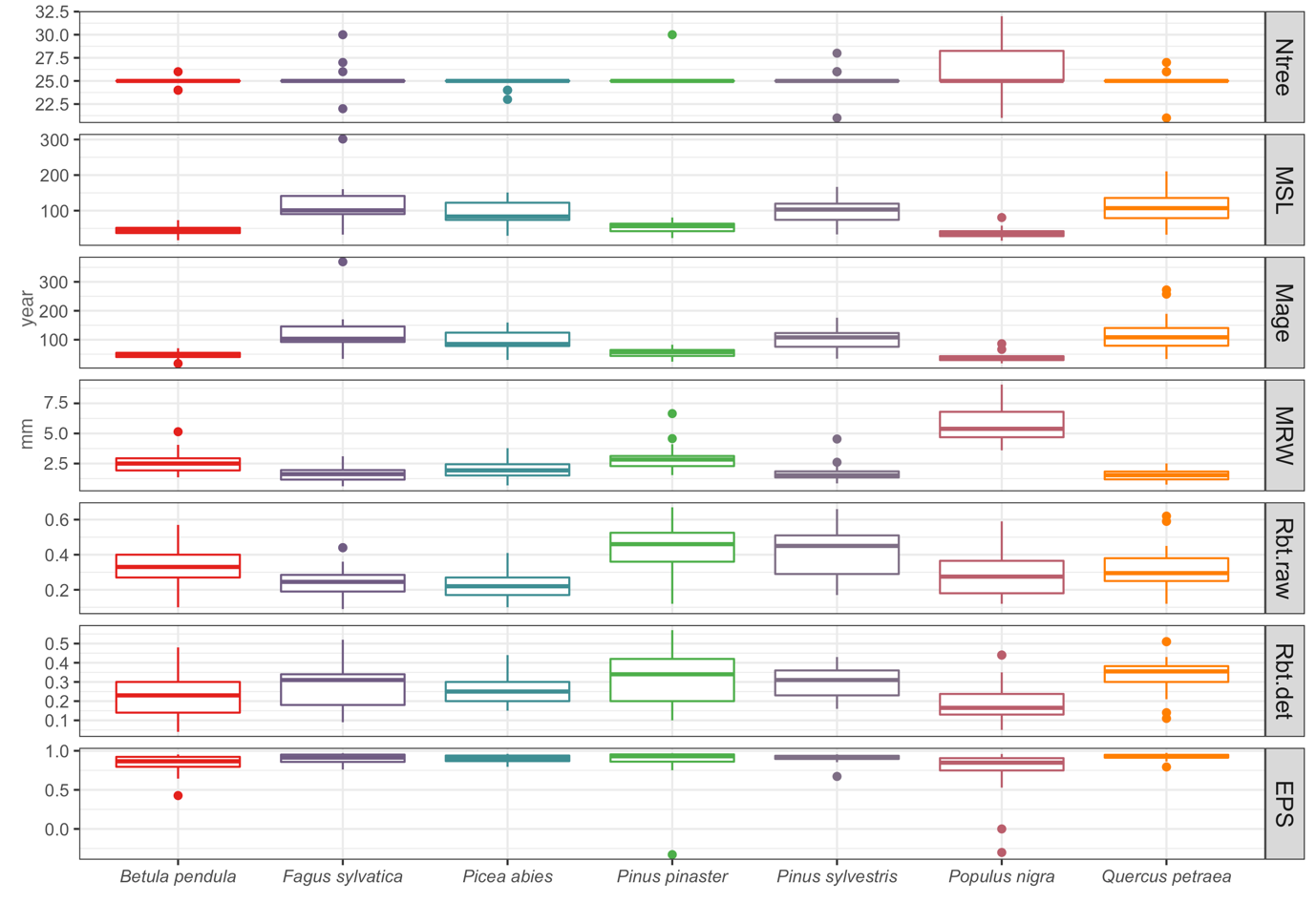


**Fig. S1.** Comparison of parameters and statistics related to ring width series among tree species. Ntree, number of trees; MSL, mean series length; Mage, mean age; MRW, mean ring width; Rbt.raw, mean intercorrelation among raw series; Rbt.det, mean intercorrelation among detrended series; EPS, expressed population signal calculated for the common period, Mdensity; mean wood density. Note that EPS and Rbt.det have been calculated after applying a 32-year spline to raw series.


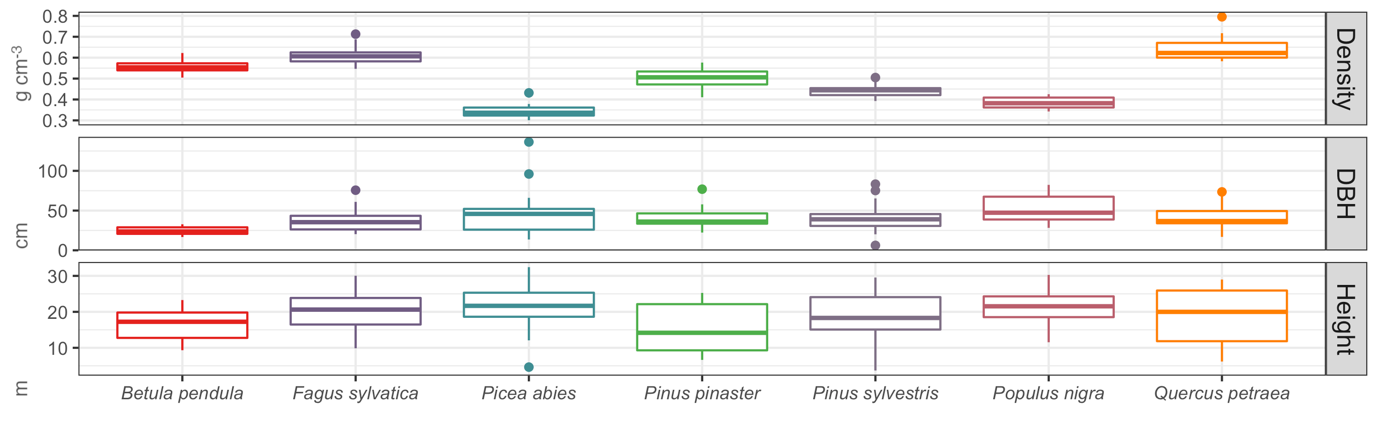


**Fig. S2.** Comparison of mean values for some tree-related parameters across tree species, obtained from the sampling sites. DBH, diameter at breast height.
